# High rates of viral suppression in a cohort of HIV-positive adults receiving ART in Ethiopian health centers irrespective of concomitant tuberculosis

**DOI:** 10.7448/IAS.17.4.19612

**Published:** 2014-11-02

**Authors:** Anton Reepalu, Taye Tolera Balcha, Sten Skogmar, Zelalem Habtamu Jemal, Erik Sturegård, Patrik Medstrand, Per Björkman

**Affiliations:** 1 Infectious Diseases Research Unit, Department of Clinical Sciences, Malmö, Faculty of Medicine, Lund University, Lund, Sweden; 2 Infectious Diseases Research Unit, Faculty of Medicine, Lund University, Ministry of Health, Addis Ababa, Ethiopia, Malmö, Sweden; 3 Oromia Regional Health Bureau, Addis Ababa, Ethiopia; 4 Clinical Microbiology, Region Skåne, Regional and University Laboratories, Malmö, Sweden; 5 Department of Laboratory Medicine, Malmö, Faculty of Medicine, Lund University, Lund, Sweden

## Abstract

**Introduction:**

Antiretroviral therapy (ART) initiation during treatment for tuberculosis (TB) improves survival in HIV/TB co-infected patients. Data on ART outcome for HIV/TB co-infected patients managed in primary health care in low-income regions is limited. We compared virological suppression rates, mortality and retention in care in HIV-positive adults receiving care in five Ethiopian health centres with regard to TB co-infection.

**Materials and Methods:**

HIV-positive ART-naïve adults eligible for ART initiation were prospectively recruited from October 2011 until March 2013. At inclusion, all patients submitted sputum for microbiological TB testing (smear microscopy, liquid culture and PCR). Virological suppression rates after six months of ART (VS; viral load <40 and <400 copies/mL) with regard to TB status was the primary outcome. The impact of HIV/TB co-infection on VS rates was determined by multivariate regression analysis. Mortality and retention in care were analyzed by proportional hazard models.

**Results:**

Among 812 participants (TB 158; non-TB 654), 678 started ART during the follow-up period (TB 135; non-TB 543). Median CD4 cell counts at ART initiation were 161 cells/µL (interquartile range [IQR], 98–243) and 184 (IQR, 118–256) for TB and non-TB patients, respectively (p=0.05). No difference in retention in care between TB and non-TB patients was observed during follow-up; 25 (3.7%) patients died and 17 (2.5%) were lost to follow-up (p=0.30 and p=0.83, respectively). Overall rates of VS at six months were 72.1% (<40 copies/mL) and 88.7% (<400 copies/mL), with similar results for subjects with and without TB co-infection (<40 copies/mL: 65/92 (70.7%) vs. 304/420 (72.4%), p=0.74; <400 copies/mL: 77/92 (83.7%) vs. 377/420 (89.8%), p=0.10, respectively). CD4 cell count increase during treatment was 87 (IQR, 26–178) and 103 cells/µL (IQR, 38–173) for TB and non-TB patients, respectively, with no significant difference between the two groups (p=0.49).

**Conclusions:**

High rates of VS were achieved in adults receiving ART at Ethiopian health centres managed by non-physician clinicians, with no significant difference with regard to TB co-infection. These findings demonstrate the feasibility of combined ART and anti-TB treatment at primary health care level in low-income countries. This study is registered with clinicaltrial.gov, NCT01433796.

